# The role of apolipoprotein E4 on calcium homeostasis in primary hippocampal neurons: an *in vitro* study

**DOI:** 10.3389/fnins.2026.1845934

**Published:** 2026-09-03

**Authors:** Deshan Liu, Yifen Zhuo, Sisi Chen, Yinzhou Wang, Xiaochun Chen, Yusheng Yao

**Affiliations:** 1 Department of Neurology, Fujian Provincial Hospital, Shengli Clinical Medical College of Fujian Medical University, Fuzhou, Fujian, China; 2 Department of Anesthesiology, Xiamen Haicang Hospital, Xiamen, Fujian, China; 3 Department of Anesthesiology, Fujian Provincial Hospital, Shengli Clinical Medical College of Fujian Medical University, Fuzhou, Fujian, China; 4 Department of Neurology, Fujian Medical University Union Hospital, Fuzhou, Fujian, China; 5 Fujian Institute of Geriatrics, Fujian Medical University Union Hospital, Fuzhou, Fujian, China; 6 Key Laboratory of Brain Aging and Neurodegenerative Disease, Fujian Key Laboratory of Molecular Neurology, Fujian Medical University, Fuzhou, Fujian, China; 7 Department of Anesthesiology, Fujian Provincial Hospital, Shengli Clinical Medical College of Fujian Medical University, Fuzhou, Fujian, China; 8 Fujian Provincial Key Laboratory of Critical Care Medicine, Fuzhou, Fujian, China

**Keywords:** ApoE4, calcium homeostasis, DAPK-1, neurotoxicity, NMDAR2B

## Abstract

**Background:**

We previously demonstrated that apolipoprotein E (apoE4) accelerates neurodegeneration in a transgenic mouse model, but the mechanism is still unclear. A previous study reported that death-associated protein kinase 1 (DAPK1) interacts with the NR2B subunit of the N-methyl-D-aspartate (NMDA) receptor, this DAPK1–NR2B interaction increases calcium intake in cells, leading to cell damage. As apoE4 is the strongest genetic risk factor for sporadic Alzheimer’s disease (sAD) and calcium dyshomeostasis is a key driver of sAD-related neurotoxicity, the unclear mechanism linking apoE4 to calcium imbalance remains a critical gap in developing targeted therapies for sAD. Addressing this gap is urgent to advance our understanding of sAD pathogenesis and identify new therapeutic targets. Our study aimed to determine whether the neurotoxic effects of apoE4 are mediated by the interaction between DAPK-1 and the NMDA receptor NR2B subunit.

**Methods:**

Primary mouse hippocampal neurons were cultured with apoEs in the presence or absence of an NMDA receptor antagonist that preferentially targets NR2B-containing receptors. Intracellular calcium concentration, cell viability and the proteins related to calcium homeostasis were detected.

**Results:**

Cells treated with apoE4 showed increased levels of intracellular calcium and cell death. The expression of DAPK-1 and NR2B was up-regulated, and interacted with each other under the condition of ApoE4 culture.

**Conclusion:**

Our study showed that apoE4 causes neurotoxicity by increasing intracellular calcium concentration. The possible mechanism of which was that apoE4 increased the expression of DAPK-1/NMDAR2B, and further promoted the interaction between the two. These results may provide new targets and ideas for the treatment of AD.

## Background

Apolipoprotein E 4 (APOE4) carrier status is the major genetic risk factor for sporadic Alzheimer’s disease (sAD). For example, a single allele increases the risk of sAD by ∼3-fold, while double allele carriers have a 15-fold risk compared to carriers of APOE3, and APOE2 is associated with an ∼2-fold decreased risk of AD per allele (Abrego-Guandique et al., 2024; Kwon and Koh, 2024). Moreover, the importance of apoE4 as a genetic risk factor for late-onset sAD have been well established (Montagne et al., 2020). We previously demonstrated that E4FAD mice exhibit considerable age-related reductions in cognitive performance and essential synaptic proteins via downregulation of the N-methyl-d-aspartate receptor (NMDAR) signaling pathway, consistent with the loss of function of apoE4 (Liu et al., 2015).

NMDARs are widely expressed in the central nervous system, and were formed with several glutamate-gated ion channels (Arrazola and Court, 2019). Functionally, NMDARs are heterotetrameric ion channels typically assembled from two obligatory GluN1 (NR1) subunits and two GluN2 (NR2A–NR2D) subunits; NR2B (GluN2B) is therefore an integral structural subunit—rather than an independent receptor—that critically determines the channel’s biophysical and pharmacological properties (Dupuis et al., 2023). Notably, these channels are highly permeable to calcium ions (Ca^2+^) (Gaidin and Kosenkov, 2023; Stewart and Chen, 2022). While NMDARs are essential for neuronal function, their excessive activation—especially of NR2B-containing subtypes—triggers a pathophysiological cascade: it increases abnormal Ca^2+^ influx, activates pro-death signaling pathways, and ultimately induces neuronal damage, a key process in AD progression (Yu et al., 2025). The activity of the NMDAR-ion channel complex is regulated by several pharmacologically distinct binding sites (Fani and Chiti, 2022), and the up-regulation of which can produce excessive cellular Ca^2+^ influx under pathological conditions, leading to cell death (Yu et al., 2025). DAPK-1 is a 160 kD protein consisting of 1,430 amino acids that contain a protein kinase domain, and it belongs to a family of calcium/calmodulin (Ca^2+^/CaM)-dependent serine/threonine kinases (Li et al., 2023). A recent study showed that DAPK-1 specifically interacts with the NMDA receptor NR2B subunit to induce the occurrence of stroke (Zhang et al., 2024).

Neurotoxicity caused by apoE4 has been reported to be associated with an increase in intracellular Ca^2+^ (Montagne et al., 2020). However, the specific molecular mechanism by which apoE4 disrupts calcium homeostasis—especially whether key signaling molecules (e.g., DAPK1, NMDAR2B) are involved—remains unclear. Although previous studies have confirmed that apoE4-induced neurotoxicity is associated with increased intracellular Ca^2+^, and DAPK1 can interact with NMDAR2B to regulate calcium influx, no research has yet clarified whether the DAPK1-NMDAR2B interaction mediates the calcium homeostasis imbalance caused by apoE4. This unclear link not only limits our understanding of apoE4-related AD pathogenesis but also hinders the development of targeted therapies for sAD. We emphasize that clarifying the mediating role of DAPK1-NMDAR2B interaction in apoE4-induced calcium dyshomeostasis is not only critical for filling the gap in understanding apoE4’s neurotoxic mechanisms in AD but also addresses an unmet clinical need—current AD therapies lack targeted strategies for apoE4 carriers, who account for the majority of sporadic AD cases. Our study thus aims to provide a mechanistic basis for developing novel targeted interventions for apoE4-related AD. In the pathophysiology of AD, apoE4-mediated upregulation of DAPK1 and NMDAR2B not only enhances their physical interaction but also amplifies NMDAR-dependent pathological Ca^2+^ influx—this process impairs mitochondrial function, increases reactive oxygen species (ROS) production, and suppresses the expression of neuroprotective factors (e.g., BDNF), ultimately exacerbating neuronal apoptosis (Yu et al., 2025). Our study targets this key pathophysiological axis to clarify its role in apoE4-induced neurotoxicity. Notably, previous studies have separately reported that apoE4 correlates with calcium dyshomeostasis, and DAPK1 interacts with NMDAR2B to regulate calcium influx. However, no prior work has directly investigated whether the DAPK1-NMDAR2B complex mediates apoE4-induced calcium imbalance—our study fills this gap by testing this specific mechanism in primary mouse hippocampal neurons, using multiple approaches (e.g., co-immunoprecipitation, calcium imaging) to confirm the interaction and its role in neurotoxicity. Above all, we hypothesized that the neurotoxic effects of apoE4 are partly mediated by the DAPK1-NMDAR2B interaction, which drives the pathophysiological process of abnormal calcium influx and subsequent neuronal death in AD. Thus, building on the unmet need to clarify the molecular link between apoE4, calcium dyshomeostasis, and AD neurotoxicity, and unlike prior studies that focused on individual factors, we examined recombinant apoE4’s effects on cell viability and calcium homeostasis in primary mouse hippocampal neurons, and used co-immunoprecipitation (to confirm DAPK1-NMDAR2B interaction) and calcium detection assays to test our hypothesis—providing direct evidence for the mediating role of this interaction.

## Materials and methods

### Cell culture

Primary hippocampal cultures were prepared from embryonic day 20 (E20) 5xFAD mice. A total of 6 pregnant E20 5xFAD mice (purchased from the Model Animal Research Center of Nanjing University) were used for all primary neuron isolations, with 3–4 embryos obtained per pregnant mouse (total 22 embryos). All pregnant mice were housed under standard conditions (12-h light/dark cycle, 22 ± 2°C, free access to food and water) prior to use. The sex of individual embryos was not determined, as reliable sex identification is not technically feasible at embryonic day 20 (E20) in mice. Neurons from embryos of both sexes were inevitably pooled; although this approach introduces sex-related biological variability, such variability was uniformly distributed across all experimental groups (apoE2, apoE3, apoE4, and control), and is therefore unlikely to introduce systematic bias into the comparative results. Animal procedures were conducted in accordance with the ARRIVE 2.0 guidelines and approved by the Animal Care and Use Committee of Fujian Medical University. Pregnant mice were randomly selected from 3 separate breeding batches to minimize batch-related variability, and embryos were randomly assigned to experimental groups (apoE2, apoE3, apoE4, control) before hippocampal dissection. Sampling steps were as follows: (1) Pregnant mice were anesthetized with 10% chloral hydrate (Sigma-Aldrich, Cat. No. C0128; 3 mL/kg, intraperitoneal injection) to ensure deep sedation. (2) Uterine horns were carefully excised under sterile conditions, and embryos were stripped from the uterus, rinsed 3 times with ice-cold, sterile Hank’s solution to remove blood and tissue debris. (3) Hippocampi were dissected from each embryo under a stereomicroscope (Olympus SZX16), with meninges and surrounding cortical tissue carefully removed using fine forceps. (4) Isolated hippocampi were transferred to ice-cold minimal essential medium (as described above) and minced into 1-mm^3^ pieces. (5) After trituration with a fire-polished Pasteur pipette, the cell suspension was filtered through a 70-μm cell strainer (BD Falcon) to remove undissociated tissue. (6) Cell viability was verified using trypan blue staining (viability >90% required for seeding), and cells were counted and seeded on poly-L-lysine-coated glass coverslips at 9 × 10^4^ cells/mL, and transferred to an incubator with a humidified atmosphere of 95% air and 5% CO_2_ at 37°C. The plating medium was exchanged with neurobasal medium (Invitrogen) containing B-27 (Invitrogen) after a 6 h incubation, and half of the medium was replaced with new media at 5 days *in vitro*. Previous studies have described more detailed protocols for cell preparation and recording techniques (Guzikowski and Kavalali, 2024).

### Cell viability

The MTT assay was used to determine whether apoE2, apoE3, apoE4, and ifenprodil differentially affected primary hippocampal neuron viability. Cells were plated at a density of 2.5 × 10^3^ cells per 100 μL per well on 96-well microtiter plates in neurobasal medium. The following day, cells were treated with apoE2, apoE3, or apoE4 (up to 0.2 μM) and ifenprodil (10 μM) in media for 48 h. The control group received an equivalent volume of vehicle (the buffer used to reconstitute the recombinant apoE proteins; e.g., PBS containing 0.1% BSA) without any apoE isoform. All groups were otherwise treated identically. For the MTT assay, 10 μL of MTT solution (2.5 mg/mL) was added to each well, and plates were incubated at 37°C for 4–8 h. The duration of MTT incubation was optimized empirically for each experimental group: cells exhibiting reduced metabolic activity (e.g., apoE4-treated neurons) required a longer incubation period (up to 8 h) to produce a formazan signal within the linear range of the assay, while cells with higher baseline metabolic activity (e.g., control neurons) reached sufficient signal within 4 h. This approach ensures that absorbance readings remain within the linear range and accurately reflect relative cell viability across groups. Then, 100 μL of solubilization solution (50% dimethylformamide and 20% sodium dodecyl sulfate [SDS], pH 4.8) was added to each well, plates were incubated for an additional 24 h, and absorption values were read at 570 nm (Liu et al., 2023).

### Intracellular calcium measurement

The fluorescence intensity of Ca^2+^ was measured by a Leica. We used standard microscopic fluo-3/AM digital imaging to measure intracellular calcium in individual hippocampal neurons (Ma et al., 2016; Shen et al., 2008; Wang et al., 2014). Briefly, hippocampal neurons were washed twice with warm (37°C) ES buffer, then loaded with 1 μM fluo-3/AM dye (Invitrogen, Cat. No. F14201) diluted in ES buffer. Cells were incubated in a 37°C, 5% CO_2_ incubator for 30 min, followed by a 15-min post-incubation in dye-free ES buffer to ensure complete de-esterification of fluo-3/AM. Live videos of selected microscopic fields were then recorded using a Leica DMI8 confocal microscope with a photomultiplier and digitized by computer with Leica imaging time course software. The somata of approximately 5–10 cells were individually measured in each microscopic field. For each biological replicate (*n* = 6 per group), 5–6 non-overlapping microscopic fields were analyzed, yielding a total of approximately 30–60 neurons measured per group. Intracellular calcium levels were estimated by converting fluorescence intensities to intracellular calcium concentrations using LAS AF Lite software. Then, hippocampal neurons were plated onto poly-D-lysine-coated culture plates and loaded with fluorescent Fluo-3/AM dye (3 μM). After washing out the unloaded paint with ES buffer (1–10 mM EGTA, 132 mM NaCl, 3.5 mM KCl, 1 mM MgCl_2_, 10 mM D-glucose, and 10 mM HEPES, pH 7.4), fluorescence was measured with a Leica fluorescence plate reader. The basal Ca^2+^ concentration was monitored for 5 min following a 1-min equilibration period; then, 10 μM ifenprodil was automatically dispensed into wells. Changes in fluorescence (F) were followed for 5 min by measuring fluorescence light emission at 538 nm using a 485 nm excitation wavelength every 20 s. Changes in cytosolic calcium ion concentration were calculated by subtracting the basal calcium levels from the averages of plateau calcium levels obtained after ifenprodil treatment (Zhou et al., 2025).

### Ca^2+^ measured by flow cytometry

Neurons were treated with apoE2, apoE3, apoE4 (0.2 μM each), or vehicle (control) for 48 h prior to calcium measurement. Hippocampal neurons were plated onto poly-D-lysine-coated culture plates and loaded with fluorescent Fluo-3/AM dye (3 μM). Intracellular calcium was measured by flow cytometry after washing out the unloaded dye with ES buffer. Flow cytometric acquisition was performed on a BD FACSCanto II flow cytometer (BD Biosciences, San Jose, CA, United States), and data were analyzed using BD FACSDiva software (version 8.0). A minimum of 10,000 events were collected per sample. F_*max*_ and F_*min*_ signals were obtained for calibration by adding ionomycin (10 μM) and EGTA (10 mM)/Triton X-100 (0.1%) to wells, respectively. Free calcium ion concentrations were calculated following the K_*d*_ of the Ca^2+^/Fluo-3 complex (345 nM). Basal calcium levels were determined as the minimal calcium ion concentration at baseline. Intracellular calcium was calculated using the following formula: [Ca^2+^]_*i*_ = K_*d*_ × (F – F_*min*_)/(F_*max*_ – F) (Kisfali et al., 2013; Li et al., 2012). All experiments were performed at room temperature (approximately 23°C).

### Immunoblotting analysis

Total cell protein (30–50 μg) was separated on a 4–12% Bis-Tris gel (Invitrogen, Cat. No. NP0321BOX) at 120 V for 90 min (Barrera et al., 2020). Proteins were then transferred to a 0.45-μm polyvinylidene fluoride (PVDF) membrane (Millipore, Cat. No. IPVH00010) using a wet-transfer system (Bio-Rad, Mini Trans-Blot^®^ Cell) at 200 mA for 120 min in transfer buffer (25 mM Tris, 192 mM glycine, 20% methanol, pH 8.3). The following primary antibodies were used: rabbit anti-NMDAR1 (1:1,000; Millipore, Cat. No. AB1516); anti-NMDAR2B (1:1,000; Millipore, Cat. No. AB1557P); anti-NMDAR2A (1:500; Millipore, Cat. No. AB1555P); rabbit anti-BDNF (1:200; Santa Cruz, Cat. No. sc-546); rabbit anti-P-CaMK-II (1:1,000; Santa Cruz, Cat. No. sc-12886); rabbit anti-p-CREB (1:1,000; Cell Signaling Technology, Cat. No. 9198); and mouse anti-DAPK-1 (1:1,000; Sigma-Aldrich, Cat. No. D1319). After incubation with an appropriate horseradish peroxidase-conjugated secondary antibody, the membranes were developed using enhanced chemiluminescence (Millipore). The X-ray film was scanned, and band densities were quantified using Image J software. The amount of protein was expressed as a relative value to β-actin.

### Coimmunoprecipitation assays

Following treatment with apoE2, apoE3, or apoE4, the growth medium was gently removed, and plates were snap cooled on ice for 15 s before gentle washing with cold phosphate-buffered saline (PBS). Cells were scraped into 1.5-mL centrifuge tubes with 1 mL of cold PBS and centrifuged at 400 × g for 4 min at 4°C to form a pellet. Cells were then lysed using coimmunoprecipitation buffer [25 mM Tris, pH 8.5, 150 mM NaCl, 1 mM EGTA, 1 mM NaF, 1 mM NaVO_4_, 3 mM beta glycerol phosphate, 1% NP40, and 5% glycerol supplemented with complete protease inhibitor (Roche)] for 10 min at 4°C with constant rotation. The lysates were then centrifuged twice at 12,000 × g for 10 min, and the pellet was discarded to ensure the complete removal of insoluble debris. Protein concentrations were determined using the Bradford method according to the kit manufacturer’s instructions (Bio–Rad).

A total of 25 μL of washed protein A/G agarose bead sediment (Pierce) was used per immunoprecipitation and added to spin columns containing paper filters (Pierce); 200 μg of 1 μg/μL protein lysate in coimmunoprecipitation buffer was added and mixed gently, and then the required antibody was added (NMDAR2B 1:1,000) before incubation at 4°C for 2 h with constant rotation. Following incubation, spin columns were spun at 200 × g for 30 s, and the unbound fraction was removed and snap-frozen for later analysis. The beads were washed 4 times with 400 μL of coimmunoprecipitation buffer for 1 min at 4°C with constant rotation. The columns were then spun at 200 × g for 30 s, and the flow-through was discarded. Protein was then eluted by the addition of 50 μL Lamelli sample buffer (5% SDS, 0.5 M DTT, 0.04% bromophenol blue, and 0.5 M Tris-HCl, pH 6.8) with heating to 95°C for 5 min. Finally, the samples were centrifuged at 1,000 × g for 2 min, and the precipitates were analyzed by western blotting as described above.

### Immunofluorescence

After treatment with apoE2, apoE3, or apoE4, hippocampal neurons were washed 3 times with PBS and then incubated in primary antibodies against DAPK-1 [rabbit polyclonal; 0.5 mg/mL in PBS supplemented with 2% NGS and 0.1% Triton X-100 (PBS-T); Sangon Biotech] or NMDAR2B (mouse polyclonal; 2.5 mg/mL in PBS-T; Sangon Biotech) overnight at 4°C. The cells were then washed 3 times with PBS and incubated with goat anti-rabbit secondary antibody conjugated to Alexa 562 (1:100; Molecular Probes) or mouse anti-mouse secondary antibody conjugated to Alexa 488 (1:100; Molecular Probes) for 2 h at room temperature. Finally, the cells were washed, mounted on slides with mounting medium (Vectashield, Vector Laboratories, Cat. No. H-1000), and observed with laser scanning confocal microscopy (excitation at 562, 488, and 405 nm to visualize the immunoreactivity of DAPK-1, NMDAR2B, and DAPI, respectively; Zeiss).

After imaging with laser scanning confocal microscopy, the density of fluorescent staining (for DAPK-1 and NMDAR2B) was quantified using ImageJ software (Version 1.8.0, National Institutes of Health, United States). For each group, 5–6 random microscopic fields were selected, and 10–15 neurons per field were analyzed. The average fluorescence intensity (MFI) of the target proteins in the neuronal somata was measured, with background fluorescence (from non-cellular areas) subtracted to avoid interference. The relative fluorescence density was calculated by normalizing the MFI of each experimental group to that of the control group (Guo et al., 2022).

### Statistical analysis

Data are expressed as the mean ± standard error of the mean (SEM) unless otherwise specified. Statistical analyses were performed using the SPSS 10.0 software package (IBM, San Diego, CA). We chose one-way analysis of variance (ANOVA) for comparing groups because our experimental design included four independent groups (apoE2, apoE3, apoE4, and control) with no repeated measurements on the same biological sample (i.e., each of the *n* = 6 independent cell cultures was treated and measured only once, without re-sampling or re-measuring the same culture under different conditions). After significant main effects from one-way ANOVA (*p* < 0.05), we used the Bonferroni *post-hoc* test to compare specific groups (e.g., apoE4 vs. apoE2, apoE4 vs. control), as it effectively controls for type I errors caused by multiple comparisons. The sample size of *n* = 6 per group refers to six independent biological replicates (i.e., six independent cell culture preparations from separate embryos or separate platings). For the MTT assay, each replicate corresponded to one well in a 96-well plate; for flow cytometry, each replicate consisted of one sample with a minimum of 10,000 events collected; for fluorescence microscopy-based calcium imaging, 5–6 random microscopic fields were captured per replicate, with 10–15 neuronal somata quantified per field; for western blotting and co-immunoprecipitation, each replicate corresponded to one independent protein lysate preparation.

### Methodological transparency

Blinding procedures were implemented during key experimental steps. For cell viability (MTT assay) and calcium detection experiments: (1) Investigators performing cell seeding, apoE treatment, and ifenprodil administration were blinded to the experimental group labels (group information was coded by a third researcher). (2) After experiments, a different researcher (unaware of the code) analyzed the MTT absorption values, calcium fluorescence intensity, and western blot band densities via ImageJ software. The code was only decoded after all data analyses were completed to avoid observer bias.

## Results

### ApoE4 reduce cell viability of hippocampal neurons in a calcium-dependent manner

**FIGURE 1:**
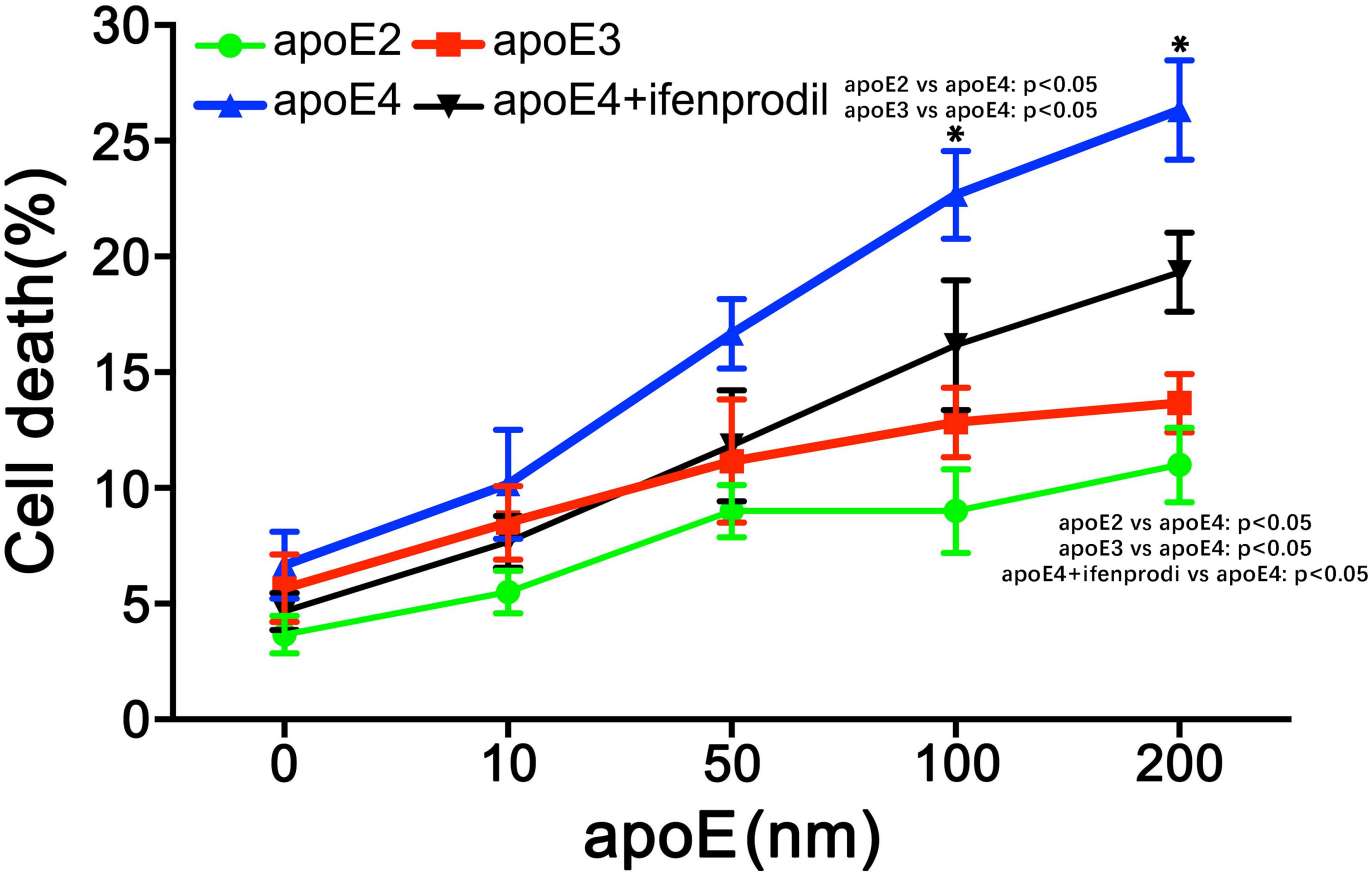
The neurotoxic effects of apoEs on hippocampal neurons. Hippocampal neurons were treated with the increasing concentrations of apoE2, apoE3, or apoE4 (0.01–0.2 μM) in the presence or absence of ifenprodil (10 μM) for 48 h, and cell viability was determined by MTT assay. Mean ± SEM is shown (*n* = 6); statistical notation: *p < 0.05 (apoE3 vs. apoE4), analyzed by one-way ANOVA followed by Bonferroni *post hoc* test. The dashed line indicates 100% cell viability (control group).

To evaluate the different effect of apoEs on hippocampal neurons, the primary cultures were treated with apoE2, apoE3, and apoE4 for 48 h. The results showed that cells treated with 0.2 μM apoE4 showed decreased cell density and eventually detached from the culture plates. In addition, MTT assay showed that apoE4 had neurotoxic effect when the concentration was > 0.05 μM, while apoE3 and apoE2 did not produce apparent neurotoxicity at various concentrations (Figure 1). These results suggested that the toxicity of apoE4 was dose- and isoform-dependent.

**FIGURE 2:**
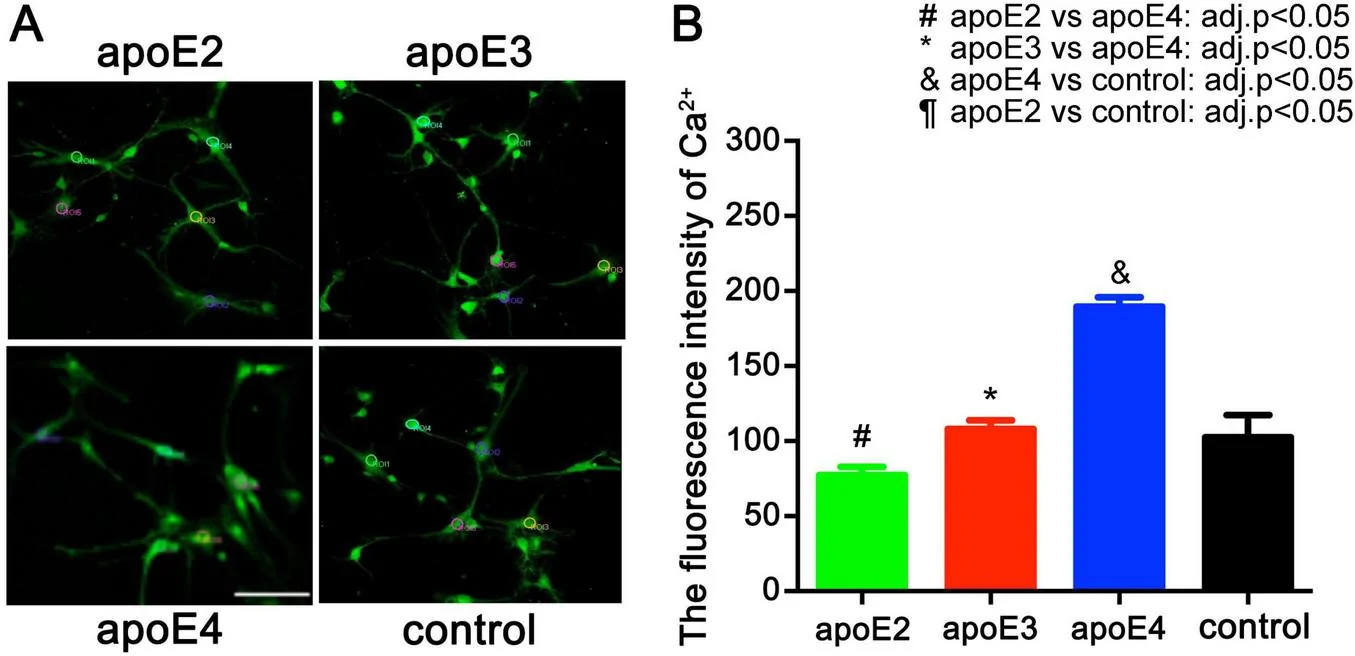
Changes of intracellular Ca^2+^ concentration in hippocampal neurons after treatment with apoEs. **(A)** The fluorescence intensity of Ca^2+^ in hippocampal neurons (scale bar = 50 μm). **(B)** Summary graph of relative Ca^2+^ fluorescence intensity in different groups (mean ± SEM, *n* = 6).

**FIGURE 3:**
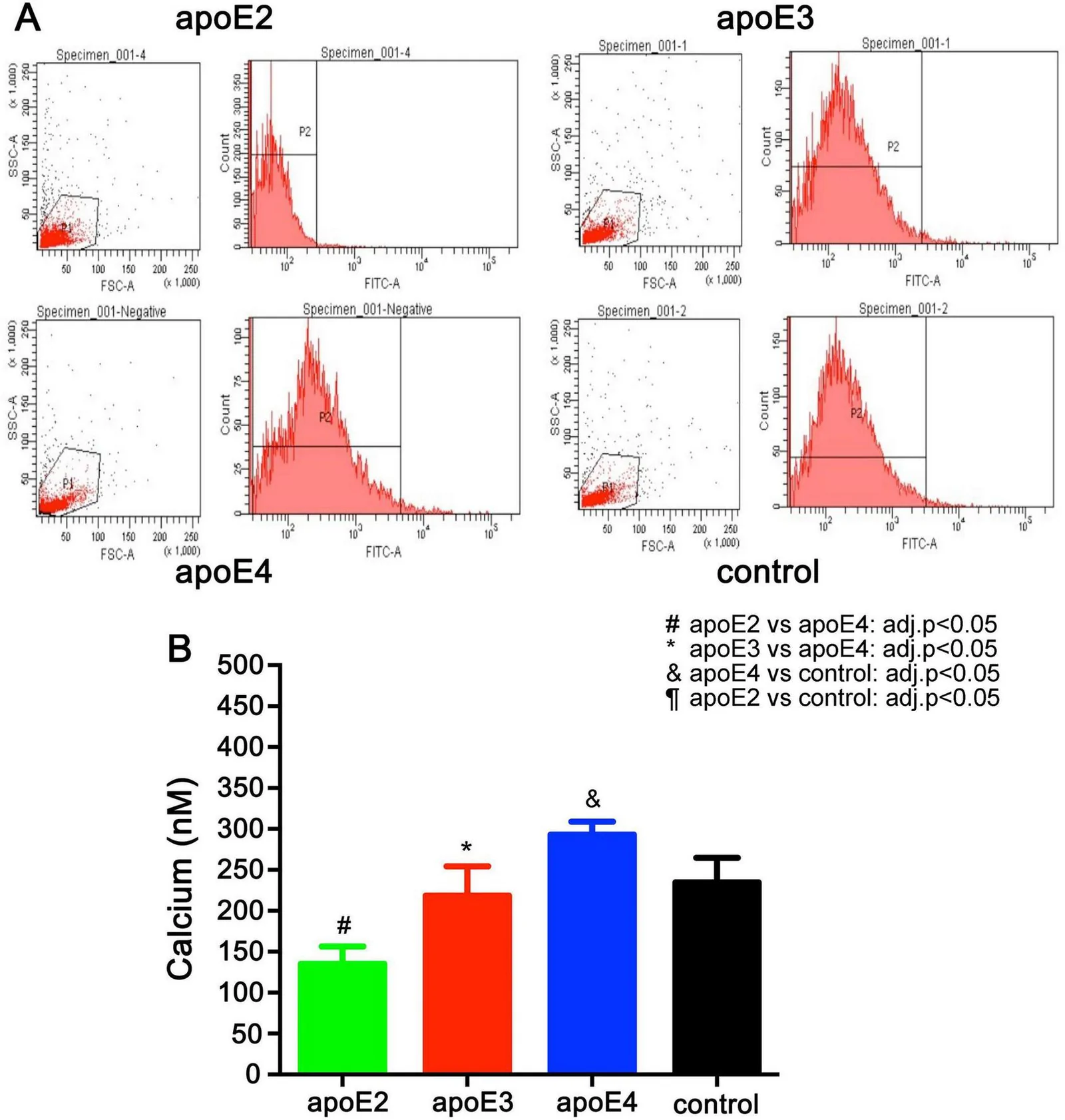
Isoform-dependent effects of apoEs on basal cytosolic calcium. Hippocampal neurons were treated with apoE2, apoE3, apoE4, or vehicle, and intracellular Ca^2+^ levels were detected using fluo-3/AM. **(A)** Representative data for intracellular calcium obtained from samples incubated for 48 h with apoE2 (0.2 μM), apoE3 (0.2 μM), apoE4 (0.2 μM), and vehicle, depicted as dot plots with the forward scatter on the horizontal axis and the ratio of fluo-3/AM fluorescence at 405 nm and 490 nm on the vertical axis. **(B)** Summary graph of Ca^2+^ concentration in different groups (mean ± SEM, *n* = 6).

To delineate the effect of apoEs on calcium homeostasis, intracellular Ca^2+^ levels was measured in hippocampal neuron cells after treatment with apoEs by using Indo-1 405/490 nm ratio and flow cytometry assays. The results showed that apoE4 increased the number of cells with high 405/490 nm ratio fluorescence compared with other groups (Figure 2). Likewise, cells treated with apoE4 increased intracellular Ca^2+^ levels compared with other groups by flow cytometry (Figure 3). In addition, ifenprodil, an NMDA receptor antagonist that preferentially targets NR2B-containing receptors, which can reduce the intracellular calcium concentration, significantly attenuates the neurotoxic effects of apoE4 (Figure 1). These results indicated that ApoE4 reduce cell viability in a calcium-dependent manner.

### ApoE4 increased the expression of NMDAR subunit in primary hippocampal neurons

**FIGURE 4:**
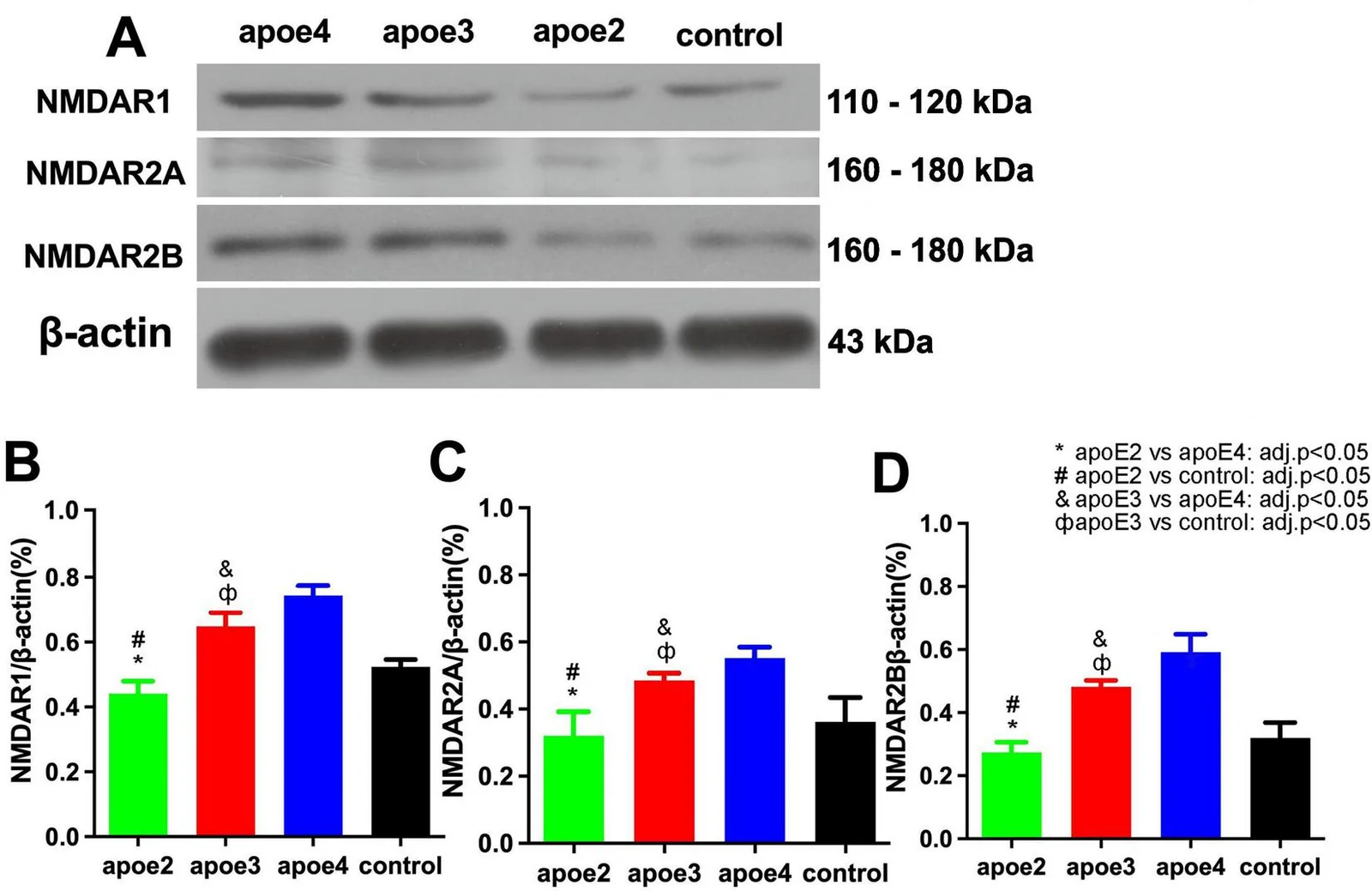
Levels of NMDAR subunit protein after treatment with apoEs. **(A)** Representative western blot images of NMDAR1, NMDAR2A, and NMDAR2B (molecular weight markers: 110–120 kDa for NMDAR1, 160–180 kDa for NMDAR2A, 160–180 kDa for NMDAR2B) with β-actin (43 kDa) as a protein loading control. Quantified relative protein levels of **(B)** NMDAR1, **(C)** NMDAR2A, and **(D)** NMDAR2B (mean ± SEM, *n* = 6). Green: apoE2, red: apoE3, blue: apoE4, and gray: control.

As the up-regulated expression of NMDAR can increase Ca^2+^ intake. Thus, we detected the expression of NMDAR subunits in hippocampal neurons under the condition of apoEs co-culture. The results showed that the expression of the 3 subunits of NMDAR in apoE4 group was higher than that in other groups (Figure 4).

### ApoE4 decreased the expression of p-CaMK-II, p-CREB, and BDNF expression in primary hippocampal neurons

**FIGURE 5:**
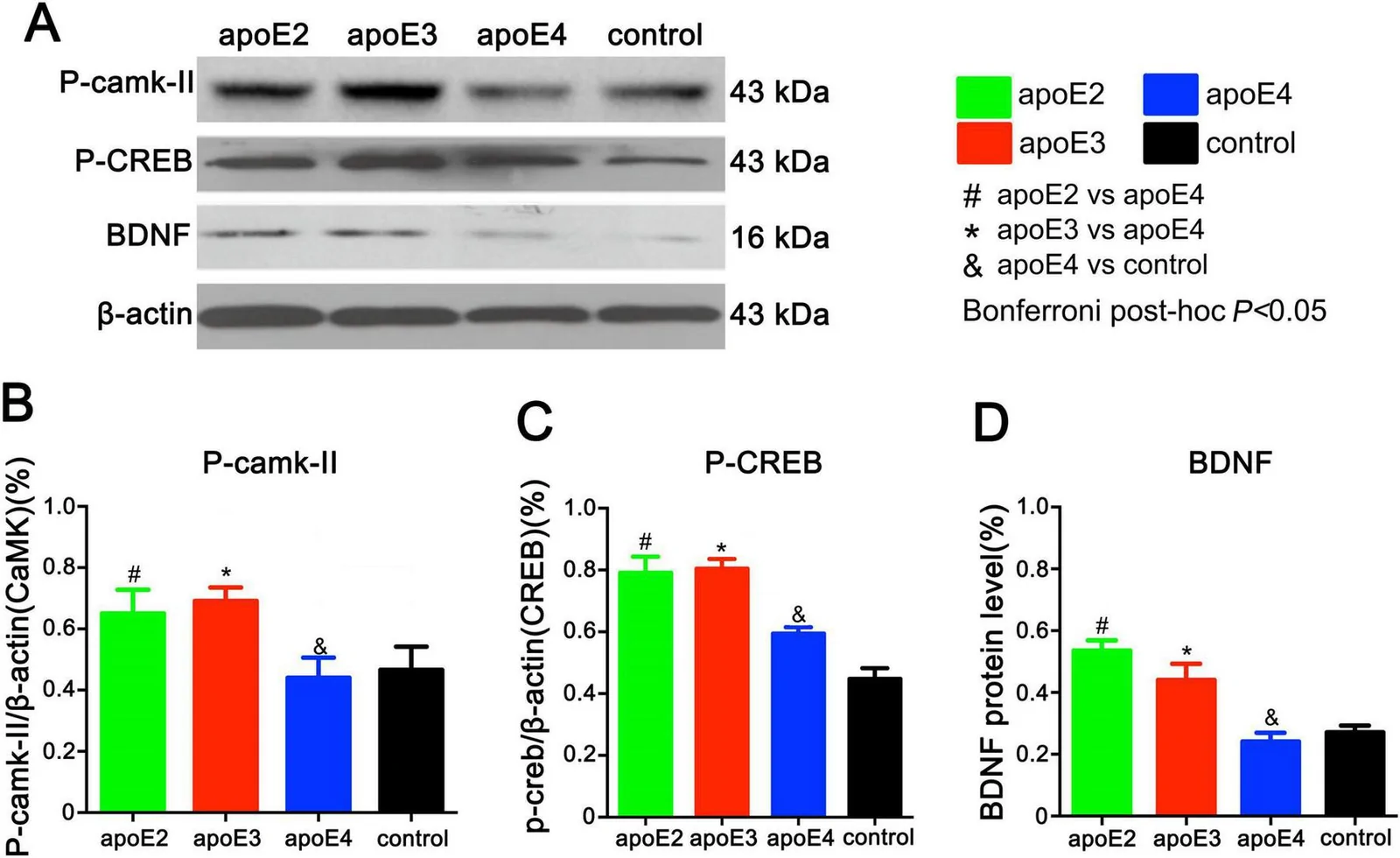
Changes of proteins related to Ca^2+^ intake pathway after treatment with apoEs. **(A)** Representative western blot images of p-CaMK-II (43 kDa), p-CREB (43 kDa), and BDNF (16 kDa), respectively, with β-actin (43 kDa) as a protein loading control. Relative protein levels of **(B)** p-CaMK-II, **(C)** p-CREB, and **(D)** BDNF (mean ± SEM, *n* = 6).

The reduced expression of CaMK-II/CREB/BDNF pathway has been reported in patients with AD and is associated with impaired neuronal function *in vitro* and *in vivo* (Cai et al., 2021; Lazaldin et al., 2023; Masmudi-Martín et al., 2022). Thus, we detected the expression of some related signaling molecules in apoEs-treated primary hippocampal neurons by western blot. The results showed that the protein levels of p-CaMK-II, p-CREB and BDNF was decreased after apoE4 treatment compared to other groups (Figure 5).

### The interaction between DAPK-1 and NMDAR2B regulates the neurotoxic effects of apoE4

**FIGURE 6:**
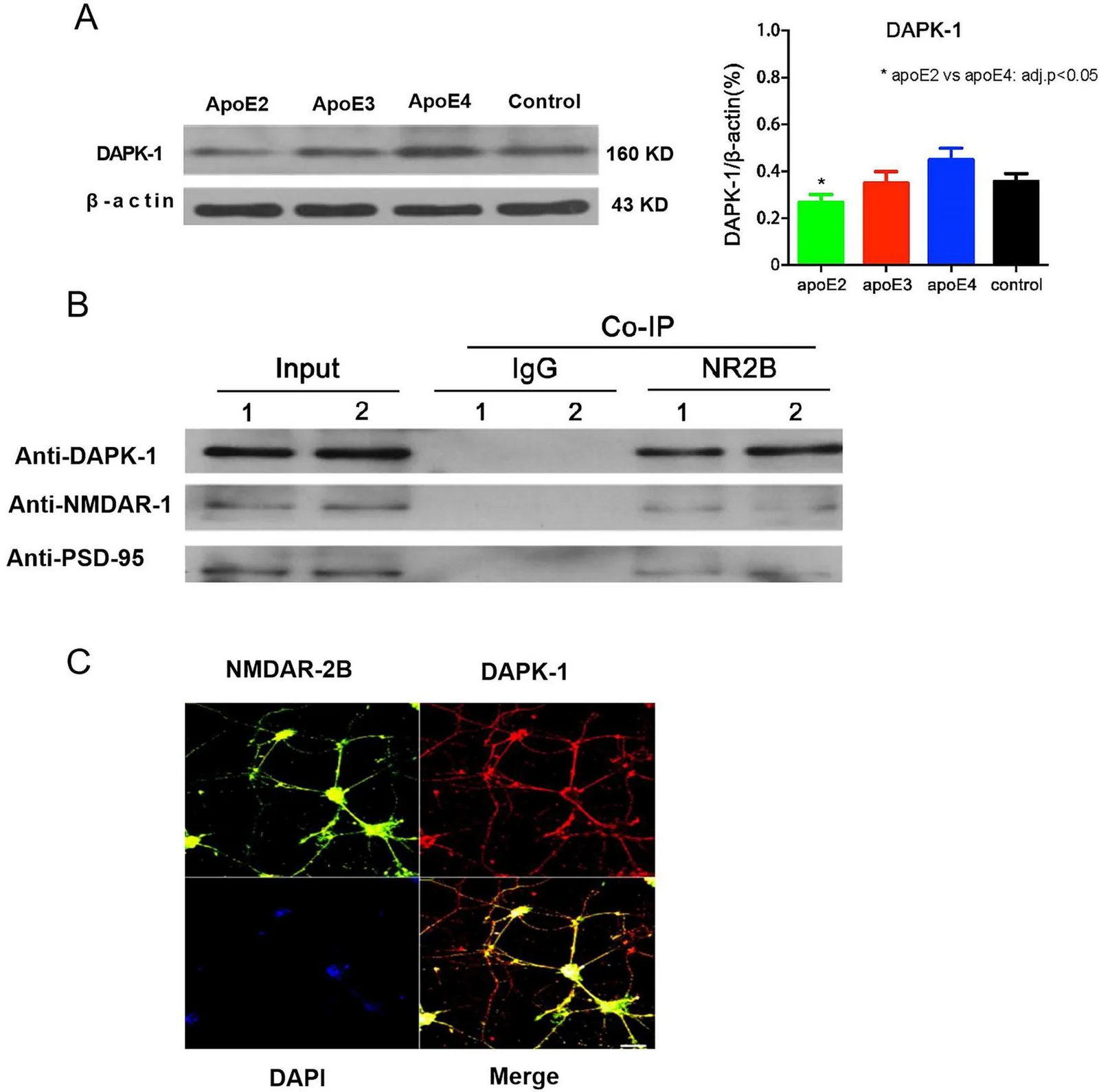
Expressions of NMDAR2B and DAPK1 were detected by co-immunoprecipitation after apoE4 treatment. **(A)** Representative western blot images of DAPK-1 (160 kDa) with β-actin (43 kDa) as a protein loading control. **(B)** Representative western blot images of protein samples immunoprecipitated with anti-NMDAR2B (NR2B) and probed for DAPK-1 (160 kDa), NMDAR2B (110 kDa), NMDAR1 (160 kDa), and PSD-95 (95 kDa). **(C)** Representative maximum-intensity projections of a hippocampal neuron stained for NMDAR2B (green), DAPK1 (red), and DAPI (nuclear stain, blue); Scale bar = 20 μm). All experiments were repeated ≥ 3 times with *n* = 6 per group.

As an integral subunit of the cell-surface NMDA receptor channel complex, NR2B does not function as an independent entity; Ca^2+^ influx attributed to NR2B-containing NMDA receptors instead occurs through activation of the assembled receptor channel itself. DAPK-1 expression was significantly increased after apoE4 treatment in hippocampal neurons compared with other groups (Figure 6). To determine whether the DAPK1-NMDAR2B protein complex is involved in Ca^2+^-induced primary hippocampal neurotoxicity mediated by apoE4, the cells were treated with 0.2 μM apoE2, apoE3, and apoE4. The results showed that NMDAR2B was bound to DAPK-1 by co-immunoprecipitation (Figure 6).

## Discussion

Our data indicated that apoE4 increased calcium intake in hippocampal neurons by regulating the expression of DAPK-1 and NMDAR2B. Some previous studies have shown that apoE 4 but not apoE 2 or apoE3 produces neurotoxicity *in vitro* (Eran and Ronit, 2022; Montagne et al., 2020) and *in vivo* (Mao et al., 2025), indicating that apoE4 plays a negative effect on neuronal integrity, consistant with our study. In addition, our data showed that peptides derived from apoE4 increase intracellular Ca^2+^ rather than apoE2 and apoE3, consistent with a recent study (Larramona-Arcas et al., 2020). Furthermore, our data are also compatible with other studies linking apoE4 to calcium homeostasis (Ramakrishna et al., 2021).

ApoE4 may indirectly increase the intracellular Ca^2+^ concentration. For example, apoE4 might affect the function or kinetics of Ca^2+^ intake via channels. The activity of the NMDAR-ion channel complex is tightly regulated by several pharmacologically distinct binding sites (Casby et al., 2024). As mentioned above, DAPK-1 interacts with the NMDA receptor NR2B subunit at extrasynaptic sites to trigger the occurrence of stroke (Yu et al., 2025). Similarly, our study showed that the neurotoxic effects of apoE4 were associated with the increased Ca^2+^ concentration, and those apoE4-treated cells showed increased DAPK-1 protein expression compared to other groups. In addition, NMDAR2B was bound to DAPK-1 by co-immunoprecipitation in our study. These results suggested that apoE4 may bind to its receptor on the cell surface and trigger a series of intracellular signaling events that increase DAPK-1/NMDAR2B expression and prompt them to bind to each other, resulting in elevated intracellular Ca^2+^ levels. Our study advances rather than reiterates prior findings: Tu et al. (2010) linked DAPK1-NMDAR2B interaction to stroke but not to apoE4 or AD, while Veinbergs et al. (2002) reported apoE4-induced calcium imbalance without identifying DAPK1-NMDAR2B as a mediator. In contrast, we are the first to demonstrate that DAPK1-NMDAR2B specifically mediates apoE4-induced calcium overload in primary hippocampal neurons from 5xFAD mice (a model that recapitulates key AD pathological features). Furthermore, we newly found that this interaction is associated with BDNF downregulation—a unique link that deepens understanding of apoE4-mediated neurotoxicity beyond previous work. Our finding that apoE4 downregulates BDNF expression is consistent with Alqahtani et al. (2025) (Alqahtani et al., 2025), who emphasized that impaired BDNF signaling is a key contributor to AD-related synaptic loss—they further noted that BDNF deficiency amplifies NMDAR-mediated calcium toxicity by reducing neuroprotective pathways. This aligns with our data showing concurrent downregulation of BDNF and upregulation of DAPK1-NMDAR2B, suggesting apoE4 may disrupt both “neuroprotective BDNF signaling” and “pathological calcium influx” to drive neuronal death. In addition, we also detected the proteins related to Ca^2+^ intake. When the expression of CREB, calmodulin-dependent protein kinase, type II (CAMK-II) and Brain-derived neurotrophic factor (BDNF) decreased, it would increase Ca^2+^ intake (Liu et al., 2021; Ni et al., 2022; Qiu et al., 2020). In our study, the expression of CREB and BDNF were down-regulated when cells were co-cultured with ApoE4, which also indicated that the intracellular calcium concentration was increased. Moreover, we used ifenprodil, an NMDA receptor antagonist that preferentially targets NR2B-containing receptors, to reduce intracellular Ca^2+^ levels, and found that the neurotoxic effect of apoE4 was dependent on intracellular Ca^2+^ concentration.

As calcium regulates some numerous neuronal signaling cascades, the calcium homeostasis would play an important role for the progression of aging and neurodegenerative diseases (Gherardi et al., 2025; Princen et al., 2024). For example, the increased cytosolic Ca^2+^ levels is a critical event in pathology of AD (Calvo-Rodriguez and Bacskai, 2021; Wang et al., 2025). Moreover, the increased cytosolic Ca^2+^ is considered to be a prerequisite for apoptosis (Galluzzi et al., 2018) and specifically the loss of neuron in AD (Samir et al., 2020; Yan et al., 2022; Yuan et al., 2019).

Overall, we found that ApoE4 increase cell death of hippocampal neurons by increasing intracellular calcium intake. Moreover, we further found that ApoE4 may lead to intracellular calcium intake by regulating the interaction of DAPK-1/NMDAR2B, which ultimately leads to cell death. Our findings may provide new therapeutic targets for the treatment of Alzheimer’s disease. Our findings hold important practical implications for Alzheimer’s disease (AD) research and clinical translation. Specifically, the DAPK1-NMDAR2B interaction we identified serves as a novel, apoE4-specific therapeutic target: considering that apoE4 carriers make up the majority of sporadic AD cases and currently lack tailored treatments, small-molecule inhibitors or peptide blockers that disrupt DAPK1-NMDAR2B binding could alleviate calcium overload and neuronal death in this high-risk population—effectively addressing a critical unmet clinical need. The therapeutic potential of targeting calcium homeostasis in AD is further supported by Al-Kuraishy et al. (2022) (Al-Kuraishy et al., 2022), who noted ursolic acid alleviates AD-related neuronal damage by restoring calcium homeostasis; while this agent acts via antioxidant pathways (distinct from our proposed DAPK1-NMDAR2B blockers), it reinforces that restoring calcium balance is a viable therapeutic strategy for apoE4-associated AD. Additionally, our results support precision medicine strategies for AD: stratifying patients by APOE genotype (i.e., apoE4 carriers vs. non-carriers) helps optimize the efficacy of therapies targeting calcium homeostasis, as our data confirm that apoE4 status directly regulates the DAPK1-NMDAR2B axis. Beyond this, our primary hippocampal neuron model provides a robust preclinical platform for screening drugs targeting the DAPK1-NMDAR2B pathway, which in turn accelerates the translation of basic mechanistic insights into clinical trials. Collectively, these implications underscore the potential of our work to advance AD treatment and reduce the global burden of apoE4-associated sporadic AD.

It is important to acknowledge potential confounding factors influencing our results. For instance, individual variability among E20 5xFAD mouse embryos (e.g., subtle developmental differences) may cause minor inconsistencies in cell viability or protein expression—we addressed this by pooling neurons from 3 to 4 embryos per culture and repeating experiments ≥ 3 times. Batch-to-batch variability in key reagents (e.g., fluo-3/AM, antibodies) might impact assay sensitivity; we minimized this by using the same reagent batch for core experiments (e.g., calcium detection, immunoblotting) and validating with positive/negative controls.

Our study has several important limitations. First, primary embryonic 5xFAD hippocampal neurons may not fully recapitulate the pathophysiology of late-onset AD, as they lack the aged microenvironment and long-term amyloid accumulation characteristic of the disease. Future studies using aged APOE4/5xFAD double-transgenic mice are needed to validate our findings *in vivo*. Second, most experiments employed a single apoE4 concentration (0.2 μM); concentration-dependent effects across a broader dose range remain to be characterized. Third, our reliance on ifenprodil as the sole pharmacological tool to implicate NMDAR2B is a limitation; complementary genetic approaches (e.g., DAPK1 siRNA knockdown) would strengthen the mechanistic conclusions. Fourth, the *in vitro* model used here does not account for glial-neuronal crosstalk or blood-brain barrier function, both of which are relevant to apoE4-mediated pathology *in vivo*. Fifth, we did not investigate the upstream mechanisms by which apoE4 regulates DAPK1 expression (e.g., via LRP1-mediated signaling), nor did we establish the causal relationships between p-CaMK-II/p-CREB/BDNF downregulation and calcium dysregulation. These remain important open questions for future investigation.

## Conclusion

Our study demonstrates that apoE4 induces neurotoxicity in primary hippocampal neurons by disrupting calcium homeostasis—specifically, apoE4 upregulates the expression of DAPK1 and NMDAR2B, promotes their physical interaction, and thereby amplifies pathological calcium influx. This finding holds direct practical relevance: as apoE4 carriers account for most sporadic AD cases and lack tailored therapies, targeting the DAPK1-NMDAR2B interaction (e.g., via small-molecule inhibitors or peptide blockers) could serve as a precision strategy to mitigate neuronal damage in this high-risk population. To advance these insights, future research should (1) validate the DAPK1-NMDAR2B axis in APOE4/5xFAD double-transgenic mice to confirm *in vivo* relevance; (2) screen for lead compounds that block DAPK1-NMDAR2B binding using our established primary neuron model; and (3) explore whether other calcium-regulating pathways (e.g., endoplasmic reticulum calcium stores) interact with this axis to modulate apoE4’s neurotoxic effects. Collectively, our work identifies a novel apoE4-specific mechanistic target and lays the groundwork for translating basic findings to AD therapeutic development.

## Data Availability

The raw data supporting the conclusions of this article will be made available by the authors, without undue reservation.
